# A Novel Autophagy-Related IncRNAs Signature for Prognostic Prediction and Clinical Value in Patients With Pancreatic Cancer

**DOI:** 10.3389/fcell.2020.606817

**Published:** 2020-12-15

**Authors:** Zhengdong Deng, Xiangyu Li, Yuanxin Shi, Yun Lu, Wei Yao, Jianming Wang

**Affiliations:** ^1^Department of Biliary and Pancreatic Surgery/Cancer Research Center Affiliated Tongji Hospital, Tongji Medical College, Huazhong University of Science and Technology, Wuhan, China; ^2^Department of Oncology Affiliated Tongji Hospital, Tongji Medical College, Huazhong University of Science and Technology, Wuhan, China; ^3^Affiliated Tianyou Hospital, Wuhan University of Science and Technology, Wuhan, China

**Keywords:** pancreatic cancer, autophagy-related lncRNA signature, prognostic prediction, LINC01559, chemoresistance

## Abstract

Autophagy is an important bioprocess throughout the occurrence and development of cancer. However, the role of autophagy-related lncRNAs in pancreatic cancer (PC) remains obscure. In the study, we identified the autophagy-related lncRNAs (ARlncRNAs) and divided the PC patients from The Cancer Genome Atlas into training and validation set. Firstly, we constructed a signature in the training set by the least absolute shrinkage and selection operator penalized cox regression analysis and the multivariate cox regression analysis. Then, we validated the independent prognostic role of the risk signature in both training and validation set with survival analysis, receiver operating characteristic analysis, and Cox regression. The nomogram was established to demonstrate the predictive power of the signature. Moreover, high risk scores were significantly correlated to worse outcomes and severe clinical characteristics. The Pearson’s analysis between risk scores with immune cells infiltration, tumor mutation burden, and the expression level of chemotherapy target molecules indicated that the signature could predict efficacy of immunotherapy and targeted therapy. Next, we constructed an lncRNA–miRNA–mRNA regulatory network and identified several potential small molecule drugs in the Connectivity Map (CMap). What’s more, quantitative real-time PCR (qRT-PCR) analysis showed that serum LINC01559 could serve as a diagnostic biomarker. *In vitro* analysis showed inhibition of LINC01559 suppressed PC cell proliferation, migration, and invasion. Additionally, silencing LINC01559 suppressed gemcitabine-induced autophagy and promoted the sensitivity of PC cells to gemcitabine. In conclusion, we identified a novel ARlncRNAs signature with valuable clinical utility for reliable prognostic prediction and personalized treatment of PC patients. And inhibition of LINC01559 might be a novel strategy to overcome chemoresistance.

## Introduction

Pancreatic cancer (PC) is one of the most lethal malignancies with a rising incidence and an extremely poor prognosis. There will be approximately 57,600 new PC cases and 47,050 deaths occurred in the United States in 2020 (Siegel et al., 2020). Although therapeutic treatments for PC have been improved, including surgery, chemotherapy, radiotherapy, and immunotherapy, 5 years survival rates remain unsatisfactory (Vincent et al., 2011; Bliss et al., 2014). Therefore, there is an urgent need to identify reliable biomarkers for the prognostic prediction and develop effective therapeutic strategies for PC patients.

Long non-coding RNA (lncRNA) is a gene transcription composed of more than 200 nucleotides, which has been reported to be aberrantly expressed and abnormally regulated in multiple cancers (Li et al., 2016; Castro-Oropeza et al., 2018). Accumulated evidence have shown that lncRNAs are involved in a variety of cancer biological processes, such as epigenetic regulation, DNA damage, immune escape, metabolic disorders, chemical resistance, as well as epithelial-mesenchymal transition (EMT), and cell stemness (Jiang et al., 2019). The underlying mechanism may be related to the mutual correction of lncRNA and other cellular molecules, including DNA, miRNA, and mRNA (Tang X. J. et al., 2019; Grixti and Ayers, 2020). At present, several lncRNAs have been identified as tumor biomarkers, such as HOTAIR, MALAT1, and H19. Iyer et al. (2015) curated a total of 7,256 RNA-seq libraries and identified 7,942 cancer-associated lncRNAs that could potentially be biomarkers for specific cancer types. Thus, better understanding of the role of lncRNAs in cancer is helpful to identify novel diagnostic biomarkers and develop potential therapeutic targets.

Autophagy, also known as type II cell death, is a process in which cells use lysosomes to degrade their damaged organelles and macromolecules under the regulation of autophagy related signaling pathways. Autophagy is involved in pathophysiological processes of multiple diseases, including neurodegenerative diseases, metabolic diseases, infectious diseases, and cancers (Yang et al., 2017). Flaks et al. (1981) first proposed the presence of autophagy during pancreatic carcinogenesis. Indeed, the role of autophagy in cancer is still controversial. Emerging evidence suggests that suppressed autophagy contributes to initiation of carcinogenesis, while activated autophagy is required for malignancy maintenance and development (Chen K. D. et al., 2018; Folkerts et al., 2019). Moreover, autophagy is reported to play a vital role in cancer cells survival, metastasis, and drug resistance (Yun and Lee, 2018). Several cellular molecules and signaling pathways are involved in autophagy regulation, including lncRNAs. Hu et al. reported that lncRNA MALAT1 regulated autophagy-related chemoresistance in gastric cancer (YiRen et al., 2017). However, the role of autophagy-related lncRNAs in PC has been not fully elaborated yet. Therefore, this study aimed to identify the autophagy-related lncRNAs and explore their clinical relevance in PC.

In the present study, we identified the autophagy-related lncRNAs of PC and established a risk model that could provide valuable clinical utility for prognostic prediction and potential drugs selection of PC patients.

## Materials and Methods

### Data Acquisition and Processing

The pancreatic adenocarcinoma RNA-seq data and corresponding clinical information were downloaded from the TCGA dataset^1^. The cohort contains 178 tumor tissues and four normal pancreatic tissues. And, 177 PC patients with complete clinical information were extracted for further analysis. Perl language was performed to convert gene names from Ensemble IDs to a profile of gene symbols with the Ensemble database^2^.

### Identification of Autophagy-Related lncRNAs

Autophagy-related genes (ARGs) were obtained from the Human Autophagy Database^3^. We extracted the lncRNA list from mRNA expression data of the GNECODE project^4^. Then, the Pearson correlation was applied to analyze the correlation between the lncRNAs and ARGs. The lncRNAs with correlation coefficient |*R*^2^| > 0.5 and *p* < 0.01 were considered as autophagy-related lncRNAs (ARlncRNAs).

### Construction and Validation of an ARlncRNAs Prognostic Signature

To increase the reliability of our study, we randomly divided the entire dataset into a training set (accounting for 60%) and a validation set (accounting for 40%) by the “caret” R package (version 6.0-84)^5^ (Deist et al., 2018). And the whole dataset was considered as an entire set (*n* = 177). At first, we adopted the univariate cox regression analysis to identify the significant ARlncRNAs in the training set with a *p* < 0.01 by the “survival” R package. Then, the least absolute shrinkage and selection operator (LASSO) penalized cox regression analysis was performed to further reduce the dimension and the multivariate cox regression analysis was utilized to calculate the risk coefficients of the prognostic signature. The risk score formula is shown as follows: Risk score = ΣCoef ARlncRNAs × Exp ARlncRNAs. The Coef ARlncRNAs represents the coefficient of each ARlncRNAs and Exp ARlncRNAs is the expression of each ARlncRNAs. Based on the median risk score of the signature, the patients were divided into low-risk and high-risk groups. The survival analysis for the different groups was performed using the Kaplan-Meier (K-M) survival curve analysis and log-rank test analysis with the “survminer” R package. Moreover, we constructed the receiver operating characteristic (ROC) curve by using the “survivalROC” R package to evaluate the specificity and sensitivity of the prognostic signature.

### The Nomogram Establishing

In order to simplify the predictive model, we created a nomogram based on independent clinical prognostic factors with the “rms” R package (Iasonos et al., 2008). We plotted the calibration curve of the nomogram to value the predictive power of the prognostic signature.

### Bioinformatics Analysis of the Signature

Grouped samples and expression patterns were analyzed using the principal component analysis (PCA). Gene set enrichment analysis (GSEA) was performed to evaluate different functional phenotypes between low- and high-risk groups (Subramanian et al., 2005). Moreover, we analyzed the correlation between different risk groups and clinical characteristics with the chi-square test and the results were presented in a heat map.

To better investigate the relationship between the signature and immune cell infiltration, we calculated the infiltration expression of 22 immune cells in PC by using the “CIBERSORT” R package. Then, the immune-related Pearson correlation coefficients were tested for relevance in the R program.

Moreover, to explore the clinical utility of the signature to predict therapeutic effect, we used the Pearson’s analysis to calculate the correlation between risk scores with molecules of targeted therapy. The therapy targets are as follows: programmed cell death 1 (PD-1, also known as PCDC1), programmed cell death ligand 1 (PD-L1, also known as CD274), epidermal growth factor receptor (EGFR), vascular Endothelial Growth Factor Receptor 3 (VEGFR3, also known as FLT4), KIT proto-oncogene (KIT), Fms-like tyrosine kinase 3 (FLT3), MET proto-oncogene (MET), vascular Endothelial Growth Factor Receptor (VEGFR1, also known as FLT1), mammalian target of rapamycin (mTOR), platelet-derived growth factor receptor alpha (PDGFRA), and platelet-derived growth factor receptor beta (PDGFRB).

### Construction of the lncRNA–miRNA–mRNA Regulatory Network

The DIANA online tools^6^ were employed to explore the miRNAs binding to lncRNA. We employed three miRNA databases, including miRDB^7^, miRTarBase^8^, and TargetScan^9^, to predict the target genes of miRNAs. To predict the expression correlation between lncRNAs and miRNAs, the threshold was set at 0.9. Subsequently, the lncRNA-miRNA-mRNA regulatory network was mapped by the Cytoscape (version 3.7.0)^10^ to better understand the connections.

### Functional Annotation and Pathway Analysis of the Target mRNAs

To further explore the functional annotation and pathway analysis of the target mRNAs, the Gene Ontology (GO) and Kyoto Encyclopedia of Genes and Genomes (KEGG) enrichment analysis were performed by using the “clusterProfiler” R package with a *p* < 0.05, and FDR < 0.05.

### Identification of Potential Small Molecule Drugs

Connectivity Map (CMap)^11^ is a collection of genome-wide transcriptional expression data from cultured human cells treated with bioactive small molecules and analyzed by corresponding matching algorithms to investigate the relationship between drug and gene expression changes and phenotypes (Lamb et al., 2006). We uploaded up- and down-regulated target genes from the lncRNA-miRNA-mRNA network to CMap. A connectivity score ranging from -1 to 1 was used to reflect the degree of closeness between the expression spectrums. The drugs with negative scores were potential therapeutic molecules. Moreover, these candidate drugs were investigated in the Pubchem database^12^.

### Gene Expression Profiling Interactive Analysis (GEPIA)

Gene Expression Profiling Interactive Analysis (GEPIA)^13^ is a website for large-scale expression analysis and interactive analysis that has been used to compare the expression of signature lncRNAs (Tang Z. et al., 2019).

### Patients and Samples

Blood samples of PC patients were collected from the Tongji Hospital, Tongji Medical College, Huazhong University of Science and Technology (Wuhan, China) between May 2019 and July 2019. Patients were eligible if they didn’t receive any preoperative radiation and chemotherapy and the postoperative pathology was officially diagnosed as pancreatic adenocarcinoma. Exclusion criteria are as follows: (1) patients with history of previous cancer; (2) patients with multiple tumors or PC is not a primary lesion; and (3) patients with co-morbidities of the blood system. Finally, 30 of the 37 blood samples were eligible for further study. And, 10 blood samples of healthy donors were collected as a control group.

The serum specimen was separated at 3,000 rpm for 10 min from the venous blood. All the serum samples were stored at −80°C. Ethical approval for the use of human samples was obtained from the Tongji Hospital Research Ethical Committee.

### RNA Extraction and Quantitative Real-Time PCR

Total RNA was extracted from serum samples and cells by the TRIzol reagent (Life Technologies, Thermo Fisher Scientific, United States). The complementary DNA was synthesized with the PrimeScript^TM^ RT Master Mix (Takara Bio Inc, Dalian, China) according to the manufacturer’s instructions. Quantitative real-time PCR (qRT-PCR) was performed using a SYBR Green PCR kit (Thermo Fisher Scientific) following the standard protocol. And GAPDH served as the internal control. The forward primer for LINC01559 was 5′-GTCCTGCAGAACTCCCTCTT-3′, the reverse primer for LINC01559 was 5′-AGTCCTGGAGCTGCAGAAAT-3′. The forward primer for AC245041.2 was 5′-TTGCCCCCATCTTTGCCATTCC-3′, the reverse primer for AC245041.2 was 5′- TTGACCCATCTTTCCTCCCCAC-3′. The forward primer for AC005332.6 was 5′-AAGACAGCACG GTGTTAAAAAG-3′, the reverse primer for AC005332.6 was 5′-TTGAATCCAGGAGGCGGAAG-3′. The forward primer for GAPDH was 5′-GGAGCGAGATCCCTCCAAAAT-3′, the reverse primer for GAPDH was 5′-GGCTGTTGTCATACTT CTCATGG-3′. The relative expression was calculated by the 2^–ΔΔCt^ method.

### Cell Culture and Transfection

Human PC cell lines PANC-1 and SW1990 were obtained from the American Type Culture Collection (Manassas, VA, United States). These cells were maintained in the Dulbecco’s modified Eagle medium (Gibco, Carlsbad, CA, United States) supplemented with 10% fetal bovine serum (FBS; Gibco), 100 units/mL of penicillin, and 100 mg/mL of streptomycin (Sigma-Aldrich, St. Louis, MO, United States). All cell lines were authenticated, mycoplasma-free and cultured at 37°C in a humidified incubator containing 5% CO_2_.

LINC01559 si-1, LINC01559 si-2 and si-NC were purchased from the DesignGene Biotechnology (Shanghai, China) and transfected into PC cells using the Lipotransfectamine 3000 (Thermo Fisher Scientific). The lentiviral vector containing the tandem-labeled GFP-mRFP-LC3 reporter were also constructed by the DesignGene Biotechnology (Shanghai, China), and transfection was carried out according to the manufacturer’s specification.

### Western Blot Analysis

Western blotting assay was performed to detect the expression of LC3B, p62, and GAPDH as previously described (Tian et al., 2018). The antibodies of LC3B (#2775, 1:200), p62/SQSTM1 (#5114, 1:500), cleaved caspase-3 (#9661, 1:1,000), cleaved PARP (#9544, 1:1,000), and GAPDH (#5174, 1:1,000) were purchased from the Cell Signaling Technology (CST, Danvers, MA, United States). And the intensity of bands was estimated by the Image J2X (National Institute of Mental Health, Bethesda, MD, United States). All experiments were repeated three times.

### Transwell Assay

For migration assay, 1 × 10^5^/mL PC cells were suspended into the upper transwell chamber of 24-well transwell plates (8 μm pore size; Corning) containing 200 μL serum-free medium, while the lower chambers were supplied with a 500 μL complete culture medium. After 48 h co-culture, the cells on the lower surface of membrane were fixed in 4% paraformaldehyde and stained with crystal violet solution. The stained cells were then counted under a Nikon light microscope (Nikon, Japan). For invasion assay, the upper transwell chambers were coated with 60 μL Matrigel matrix gel (BD Biosciences, United States). The other operations were the same as the transwell migration experiment. All experiments were repeated three times.

### Wound Healing Assay

Indicated PC cells (2 × 10^5^ cells/well) were seeded in 6-well plates to grow to 90% confluence, and then we scratched the wound with a 200 μL pipette tip across the center of the well. After washing three times with PBS, the cells were incubated in a serum-free medium at 37°C with 5% CO_2_. Wound healing was observed with the optical microscope (Nikon, Japan) at 0 and 24 h, respectively. All experiments were repeated three times.

### *In vitro* Drug Cytotoxic and Cell Proliferation Assay

A Cell Counting Kit-8 (Dojindo Laboratories Co. Ltd, Kumamoto, Japan) assay was used to evaluate cell viability. Briefly, PANC-1 and SW1990 cells were seeded in 96-well plates at a density of 2 × 10^3^ cells per well. Each group had triplicates (*n* = 3). Cells were treated with gemcitabine at indicated concentration after 48 h. At the indicated time point, 10 μL of CCK-8 solution was added into each well and cells were incubated for 2 h at 37°C with 5% CO_2_. Then, the absorbance was measured at 450 nm with a plate reader (Bio-Tek Elx 800, United States). To assess cell proliferation, PC cells (1 × 10^3^ cells/well) were placed into 96-well plates and measured at 0, 24, 48, 72, 96. All experiments were repeated three times.

### Cell Apoptosis Assay

The percentage of apoptotic cells was analyzed by the PE Annexin V Apoptosis Detection kit (BD Pharmingen). Briefly, 1 × 10^5^/mL cells indicated PC cells were seeded into six-well culture plates. After 48 h of relevant treatment, the cells were harvested using trypsin without EDTA and washed twice with binding buffer. Finally, the cells were resuspended in 100 μL of binding buffer containing 5 μL Annexin V-PE and 5 μL 7-ADD for 15 min in the dark at room temperature, and the apoptosis was detected by flow cytometry (BD Biosciences, Franklin Lakes, NJ, United States). All experiments were repeated three times.

### Colony Formation Assay

Indicated PC cells (1 × 10^3^ cells/well) were seeded into 6-well plates and cultured in completed medium at 37°C with 5% CO_2_ for 2 weeks. Then, the cells were cleaned, fixed and dyed, and finally photographed. All experiments were repeated three times.

### Immunofluorescence and Confocal Microscopy

Cells transfected with GFP-mRFP-LC3B were grown on glass coverslips. Following the indicated treatments, cells were fixed with 4% formaldehyde for 30 min and photographed under a confocal microscope (Carl Zeiss, Germany, LSM710).

### Statistical Analysis

All statistical analysis and plotting were performed in the R language (Version 3.6.2). Univariate and multivariate Cox regression analyses were used to identify independent prognostic factors for PC. And *p* < 0.05 was considered statistically significant.

## Results

### Identification of Autophagy-Related lncRNAs (ARlncRNAs)

A total of 13,482 lncRNAs was extracted from the TCGA dataset, 825 of which were identified as ARlncRNAs by the Pearson correlation analysis (|*R*| > 0.5, *p* < 0.01).

### Establishment and Verification of a Prognostic ARlncRNAs Signature

**FIGURE 1:**
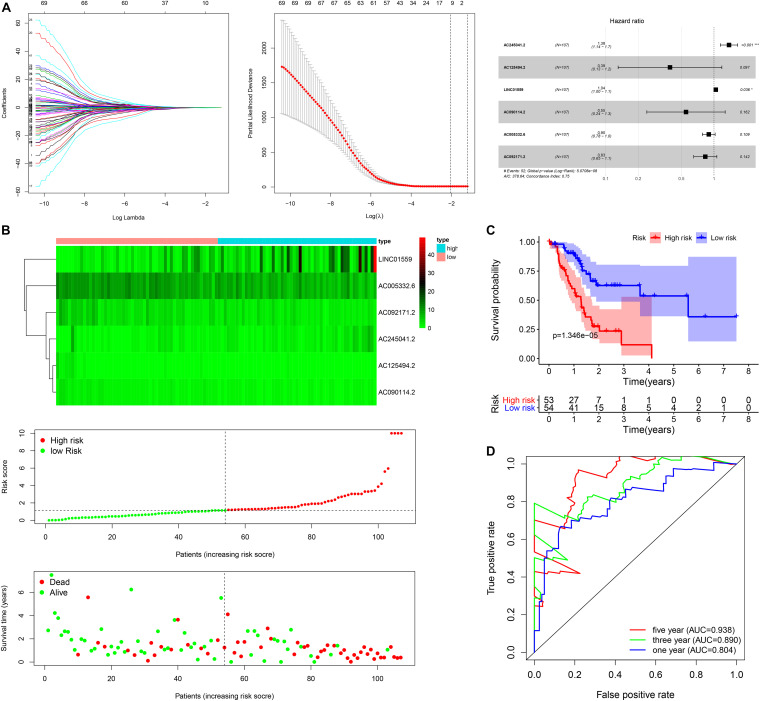
Construction of the autophagy related lncRNAs signature in training cohort. **(A)** The procedure of the construction of the SRGs signature, including univariate Cox regression analysis (left), LASSO penalized Cox regression analysis (middle) and multivariate Cox regression analysis (right). **(B)** Correlation between the prognostic signature and the overall survival of patients. The distribution of selected ARlncRNAs (upper), risk score (middle) and survival time (below). **(C)** Kaplan-Meier survival analysis of high-risk and low-risk risk group. **(D)** Receiver operating characteristic (ROC) curves for 1, 3, and 5 years survival (*p < 0.05; ***p < 0.001; ns, not significant).

Firstly, all patients were divided into training cohort (*n* = 107) and validation cohort (*n* = 70) (Table 1). Then, we employed the LASSO penalized cox regression analysis by the training cohort and found 10 more representative ARlncRNAs: AC245041.2, AL354892.2, FLVCR1.DT, AC125494.2, AL162274.2, LINC01559, AC090114.2, SH3PXD2A.AS1, AC005332.6, and AC092171.2 (Figure 1A). Moreover, the stepwise multivariate Cox regression was utilized to establish a predictive signature for PC patients in the training cohort with a risk score = (0.319702425 × expression level of AC245041.2) + (−0.934877496 × expression level of AC125494.2) + (0.038664123 × expression level of LINC01559) + (−0.594425726 × expression level of AC090114.2) + (−0.110425977 × expression level of AC005332.6) + (−0.184537572 × expression level of AC092171.2) (Figure 1A).

**TABLE 1:** Clinical information of pancreatic cancer patients in the training, validation, and entire cohort.

Variable	Entire cohort (*n* = 177)	Training cohort (*n* = 107)	Validation cohort (*n* = 70)	*p*-value
**Age**				
≤ 65	93(52.54%)	53(49.53%)	40(57.14%)	0.4023
> 65	84(47.46%)	54(50.47%)	30(42.86%)	
Gender				
Female	80(45.2%)	52(48.6%)	28(40%)	0.3324
Male	97(54.8%)	55(51.4%)	42(60%)	
Grade				
G1–2	125(70.62%)	76(71.03%)	49(70%)	0.4866
G3–4	50(28.25%)	29(27.1%)	21(30%)	
GX	2(1.13%)	2(1.87%)	0(0%)	
**Stage**				
I–II	167(94.35%)	102(95.33%)	65(92.86%)	1
III–IV	7(3.95%)	4(3.74%)	3(4.29%)	
Unknown	3(1.69%)	1(0.93%)	2(2.86%)	
**T stage**				
T1–2	31(17.51%)	22(20.56%)	9(12.86%)	0.3011
T3–4	144(81.36%)	85(79.44%)	59(84.29%)	
Unknown	2(1.13%)	0(0%)	2(2.86%)	
**M stage**				
M0	79(44.63%)	48(44.86%)	31(44.29%)	0.9106
M1	4(2.26%)	2(1.87%)	2(2.86%)	
MX	94(53.11%)	57(53.27%)	37(52.86%)	
**N stage**				
N0	49(27.68%)	33(30.84%)	16(22.86%)	0.1963
N1	123(69.49%)	72(67.29%)	51(72.86%)	
NX	5(2.82%)	2(1.87%)	3(4.29%)	

Next, the patients in the training cohort were divided into high-risk group and low-risk group based on the median risk score. Figure 1B showed the distribution of prognostic signature, survival outcomes of PC patients in different groups, and the expression profiles of the selected lncRNAs. Notably, the Kaplan-Meier survival analysis in the training cohort revealed that the survival time of PC patients was significantly longer in the low-risk group than the high-risk group (Figure 1C). As shown in Figure 1D, the area under the ROC (AUC) for 1, 3, and 5 years of the survival were 0.938, 0.890, and 0.804 in the training cohort, suggesting that the signature exerted a certain potential property for prognostic prediction in PC patients.

**FIGURE 2:**
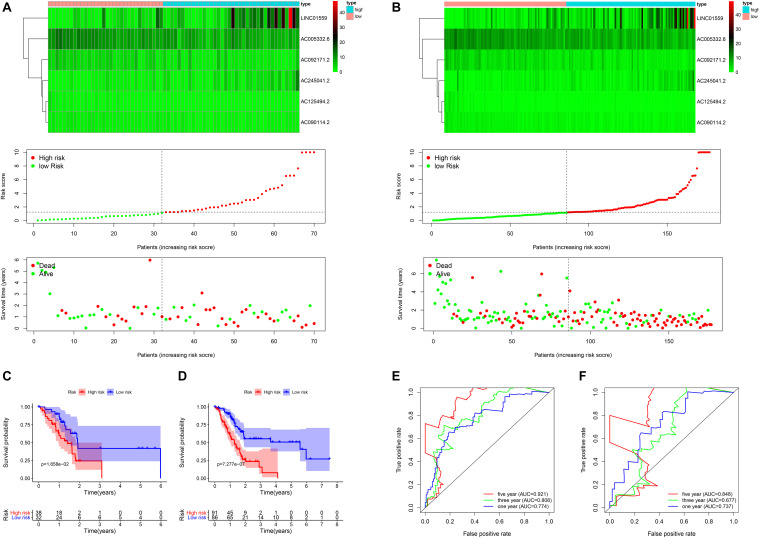
The validation of the risk model in the validation cohort and entire cohort. **(A,B)** Correlation between the prognostic signature and the overall survival of patients in the validation set **(A)** and entire set **(B)**. The autophagy related lncRNAs levels (upper), risk scores (middle), survival time (below). **(C,D)** Kaplan–Meier curves of overall survival in the training cohort **(C)** and the validation cohort **(D)** stratified by risk scores. **(E,F)** Receiver operating characteristic (ROC) curve analysis for the survival prediction model in the training cohort **(E)** and the validation cohort **(F)**.

To verify the accuracy of the signature, we analyzed its prognostic value in the validation cohort and entire cohort. LncRNAs expression profiles, risk distribution, and survival rate in the validation cohort and entire cohort were shown in Figures 2A,B. Similar to the results in the training cohort, the Kaplan-Meier (K-M) survival analysis in both the validation cohort and entire cohort indicated that the survival outcome of PC patients was better in low-risk group than in high-risk group (Figures 2C,D). The AUC at 1, 3, and 5 years were and 0.848, 0.677, 0.737, and 0.921, 0.808, 0.774 in the validation cohort and entire cohort, respectively (Figures 2E,F).

**FIGURE 3:**
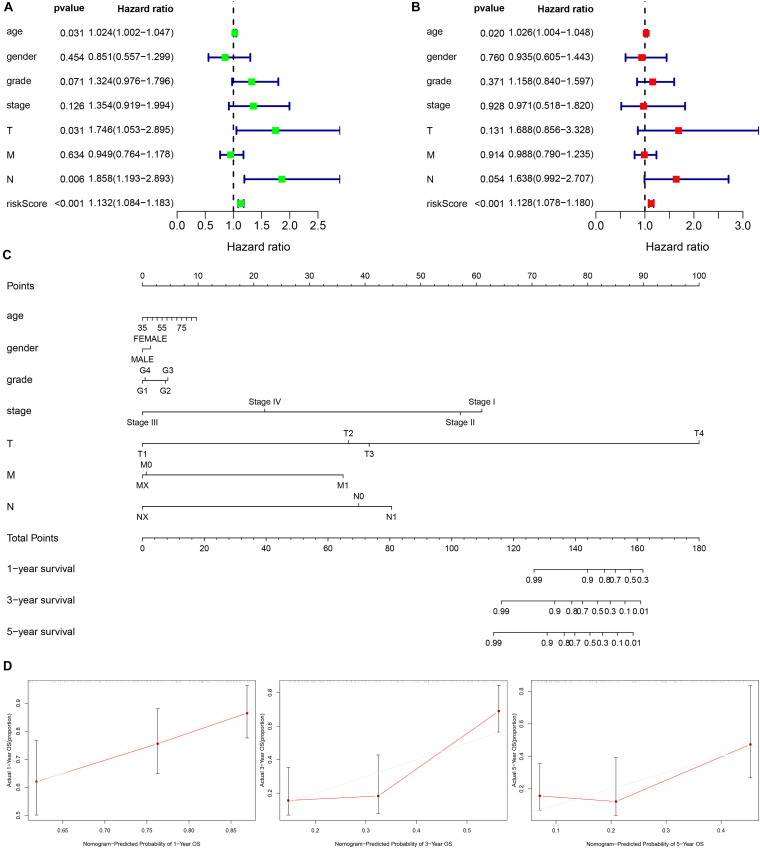
Establishment of a nomogram based on clinical characteristics and the autophagy related lncRNAs signature. **(A,B)** Univariate Cox regression analysis **(A)** and Multivariate Cox regression analysis **(B)** of clinical features and the signature. **(C)** The nomogram consists of clinical characteristics and prognostic signature. **(D)** The nomogram calibration curve is used to predict 1, 3, and 5 years survival rates.

Furthermore, the univariate and multivariate Cox regression analysis were employed to confirm the independent prognostic role of the signature for PC patients in the entire cohort (Figures 3A,B). Another independent prognostic factor was age. Moreover, a nomogram based on the signature risk score and clinical features was constructed and the calibration curve for 1, 3, and 5 years of the nomogram showed a great predictive power of the prognostic signature (Figures 3C,D).

### Functional Analysis of the Signature

**FIGURE 4:**
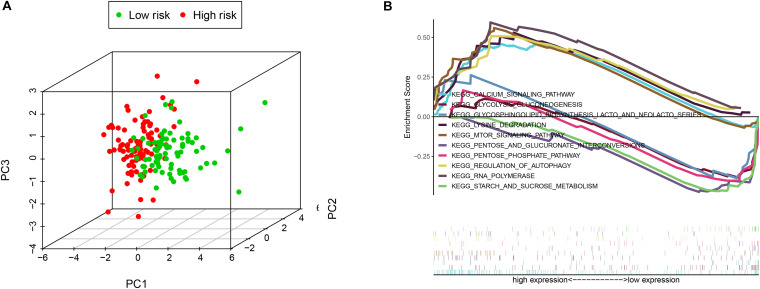
The low-risk and high-risk groups displayed different autophagy statuses. **(A)** Principal components analysis between low- and high-risk groups on the basis of the autophagy-related gene sets. **(B)** Gene set enrichment analysis (GSEA) of the two groups.

PCA was employed to demonstrate the significant distribution difference between low- and high-risk groups based on the risk scores (Figure 4A). Then, GSEA was implemented to explore the significant enriched pathways between the two groups. As shown in Figure 4B, the top five up-regulated and down-regulated KEGG pathways were the “mTOR signaling pathway,” “calcium signaling pathway,” “regulation of autophagy,” “RNA polymerase,” “lysine degradation,” and “pentose phosphate pathway,” “starch and sucrose metabolism,” “glycolysis gluconeogenesis,” “glycosphingolipid biosynthesis lacto and neolacto-series,” and “pentose and glucuronate interconversions,” respectively. These results indicated that the high-risk score was significantly associated with autophagy regulation and several signaling pathways may participate in the process.

### The Relationship Between the Signature and the Clinical Features in PC

**FIGURE 5:**
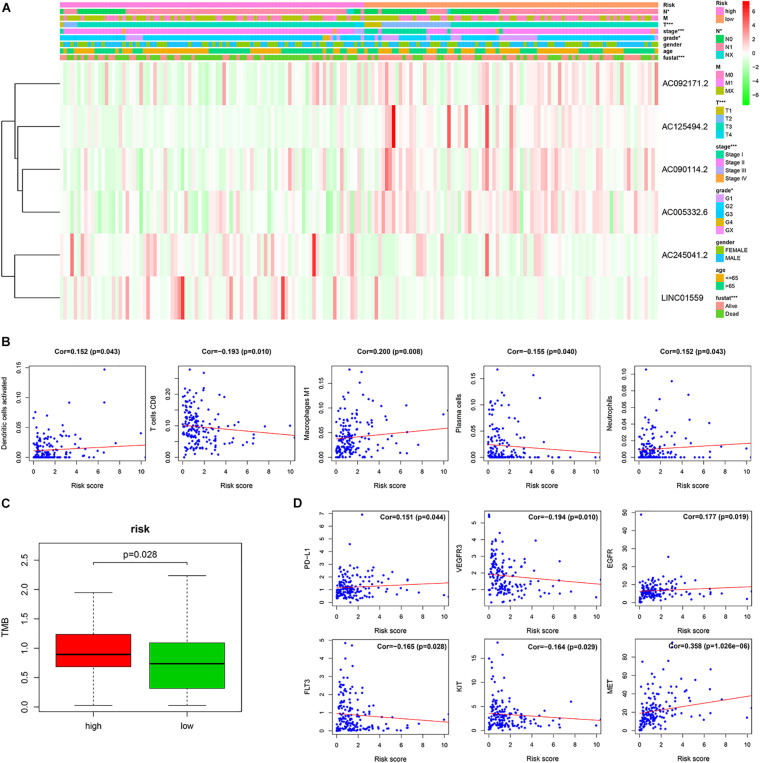
The correlation of the risk score with the clinical traits, immune cells and therapy targets. **(A)** Correlation of risk group and clinical traits. **(B)** The correlation between the risk score and immune cells. **(C)** The TMB of PC patients in the high-risk and low-risk groups. **(D)** The correlation between the risk score and therapy targets (*p < 0.05; ***p < 0.001; ns, not significant).

To investigate the clinical utility of the signature, we explored the relationship of the signature with clinical features. We found that the high-risk score was significantly correlated with tumor grade, AJCC stage, N stage, T stage, and survival status (Figure 5A).

### The Relationship of the Signature and Immune Cell Infiltration in PC Tissues

To investigate the relationship between the prognostic signature and immune cell infiltration. Pearson correlation analysis showed that the signature score was significantly correlated with the infiltration of activated dendritic cells (cor = 0.152, *p* = 0.043), plasma cells (cor = −0.155, *p* = 0.040), CD8 T cells (cor = −0.193, *p* = 0.010), M1 macrophages (cor = 0.200, *p* = 0.008), and neutrophils (cor = 0.152, *p* = 0.043) (Figure 5B).

### Predicting Efficacy of Immunotherapy and Targeted Therapy With the Signature

The tumor mutation burden (TMB) has been shown to be related to the clinical efficacy of immunotherapy (Samstein et al., 2019). To explore the value of our signature for predicting the efficacy of immunotherapy in PC, we assessed the TMB of PC patients in the high-risk and low-risk groups. We found that TMB of PC patients in the high-risk group was higher than that in the low-risk group, which implied that immunotherapy may be a potentially effective treatment to those PC patients with high-risk scores (Figure 5C).

Furthermore, we analyzed the correlation of the signature score with the therapy-related targets. Pearson’s correlation analysis showed that the risk score was significantly associated with PD-L1 (cor = 0.151, *p* = 0.044), VEGFR3 (cor = −0.194, p = 0.010), EGFR (cor = 0.177, *p* = 0.019), FLT3 (cor = −0.165, *p* = 0.028), KIT (cor = −0.164, *p* = 0.029), and MET (cor = 0.358, *p* = 1.026e–06) (Figure 5D).

### Construction of the lncRNA–miRNA–mRNA Regulatory Network

**FIGURE 6:**
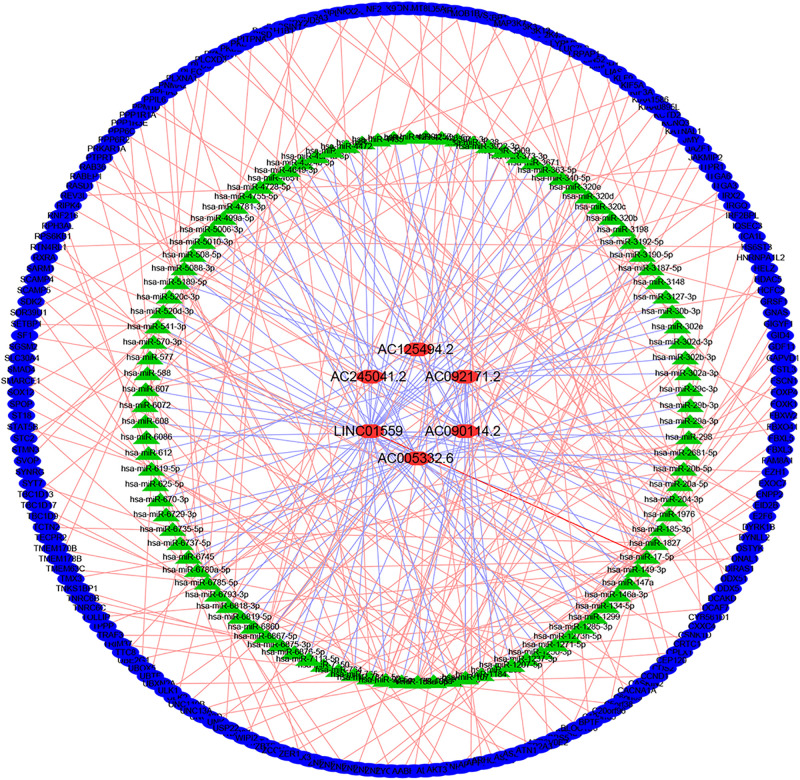
Construction of lncRNA-miRNA-mRNA regulatory networks.

LncRNAs could interact with miRNAs to modulate mRNA expression, thereby modulating the biological characteristics of malignant tumors. To explore the regulation of these selected LncRNAs, we constructed a regulatory network consisting of six lncRNAs, 107 miRNAs, and 209 mRNAs (Figure 6).

### Functional Analysis of the Regulatory Network

**FIGURE 7:**
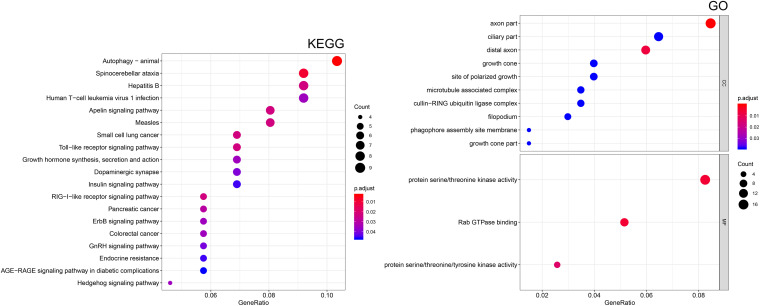
Functional enrichment analysis of target mRNAs. Kyoto Encyclopedia of Genes and Genomes (KEGG) pathway enrichment analysis of mRNAs. Gene ontology (GO) enrichment analysis of the mRNAs. CC, cellular component; MF, molecular function.

To better understand the function of the regulatory network, the “clusterProfiler” R package was employed to conduct a KEGG pathway and GO enrichment analysis. As shown in Figure 7, these genes in the regulatory network are enriched in many cellular components (CC) and molecular functions (MF). The most significantly enriched molecular functions included “protein serine/threonine kinase activity,” “Rab GTPase binding,” and “protein serine/threonine/tyrosine kinase activity,” In terms of KEGG pathway, the main significant pathways included “autophagy,” “small cell lung cancer,” “Toll-like receptor signaling pathway,” “pancreatic cancer,” “ErbB signaling pathway,” “colorectal cancer,” “endocrine resistance,” and “hedgehog signaling pathway.” These results indicated that the regulatory network may contribute to therapeutic resistance of PC through multiple signal pathways.

### Potential Small Molecule Drugs Screening

**FIGURE 8:**
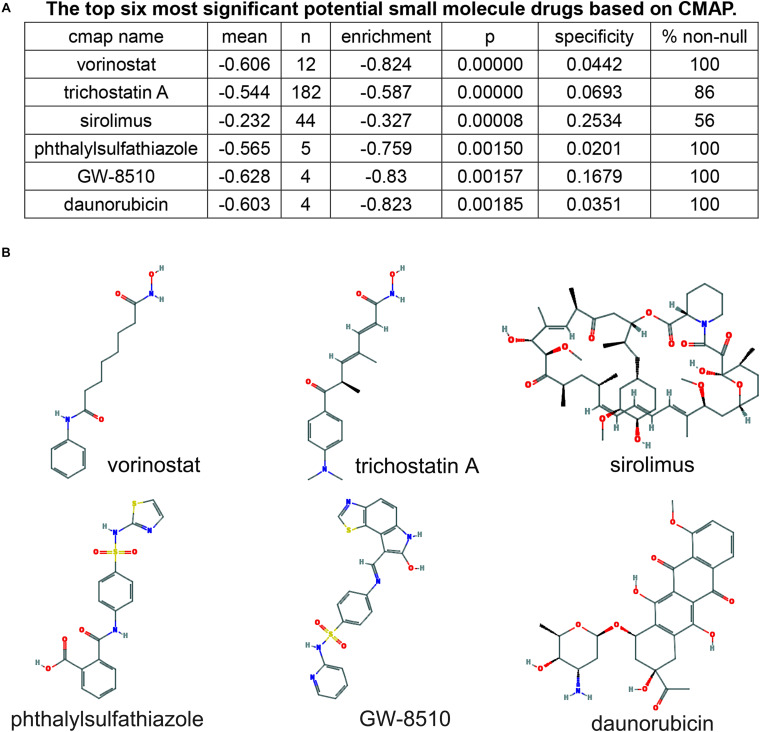
Screening of six pancreatic cancer candidate small molecule drugs. **(A)** The top six most significant potential small molecule drugs based on cMAP. **(B)** The chemical structure depiction of the six candidate small molecule drugs for PC.

To screen small molecule drugs, 209 selected mRNA were further analyzed in the Connectivity Map (CMap). The top six most significant potential small molecule drugs were listed in Figure 8A, including vorinostat (C_1__4_H_2__0_N_2_O_3_), trichostatin A (C_1__7_H_2__2_N_2_O_3_), sirolimus (C_5__1_H_7__9_NO_13_), phthalylsulfathiazole (C_1__7_H_1__3_N_3_O_5_S_2_), GW-8510 (C_2__1_H_1__5_N_5_O_3_S_2_), and daunorubicin (C_2__7_H_2__9_NO_10_). And the 2D chemical structures of these potential agents were shown in Figure 8B.

### Serum LINC01559 Served as a Diagnostic Biomarker

**FIGURE 9:**
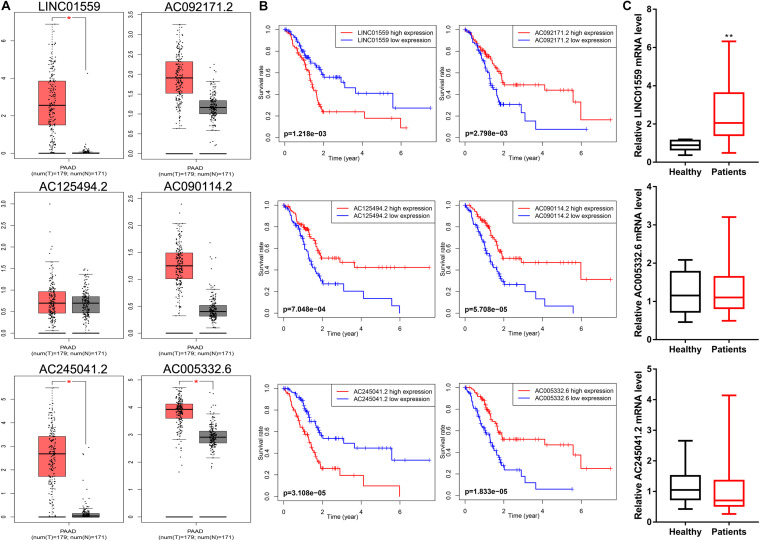
LINC01559 serves as a diagnostic biomarker. **(A)** Comparison of differential expression of signature lncRNAs by GEPIA. **(B)** Kaplan-Meier survival curves for the signature lncRNAs. **(C)** qRT-PCR was conducted to detect the expression levels of LINC01559, AC245041.2, and AC005332.6 in serum (*p < 0.05; **p < 0.01; ns, not significant).

Firstly, we evaluated the expression profiles and prognostic performance of these six lncRNAs. The expression level of AC245041.2, LINC01559, and AC005332.6 was significantly upregulated in PC than in normal tissues (Figure 9A). Moreover, the Kaplan-Meier survival analysis demonstrated the prognostic power of these six lncRNAs (Figure 9B). Then, qRT-PCR was applied to value the expression level of these lncRNAs in serum. Notably, only the expression of LINC01559 was markedly increased in the serum of PC patients, indicating that LINC01559 could serve as a diagnostic biomarker (Figure 9C).

### Inhibition of LINC01559 Suppressed PC Cell Proliferation, Migration, and Invasion

**FIGURE 10:**
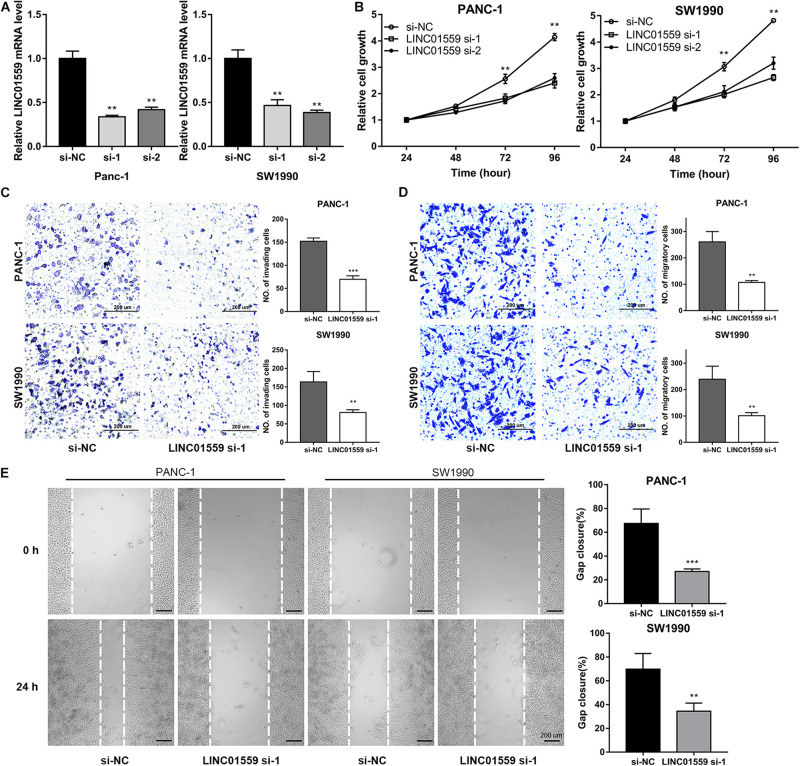
Inhibition of LINC01559 suppresses PC cell proliferation, migration and invasion. **(A)** qRT-PCR was performed to measure the expression level of LINC01559 in PC cells transfected with si-NC or two siRNA targeting LINC01559. **(B)** CCK-8 analysis was used to examine the proliferation of PC cells. **(C,D)** Transwell assays were conducted to evaluate the effect of silencing LINC01559 on PC cell invasion ability **(C)** and migratory capacity **(D)**. Scale bar: 200 μm (200×). **(E)** Wound healing assays showing the capacity of indicated PC cell migration. Scale bar: 200 μm (40×) (**p < 0.01; ***p < 0.001; ns, not significant).

We selected LINC01559 for further analysis. As shown in Figure 10A, we successfully silenced the expression level of LINC01559 in PC cells by si-LINC01559 transfection. Next, we explored the effect of silencing LINC01559 on the PC cells proliferation, migration, and invasion. CCK8 assay showed that the inhibition of LINC01559 led to a reduced viability in the PANC-1 and SW1990 cells (Figure 10B). Also, transwell assay was performed to demonstrate that the invasion and migration ability of PC cells were suppressed under LINC01559 depletion (Figures 10C,D). Furthermore, it was proved by wound healing assays that silencing LINC01559 obviously hindered the migration ability of PANC-1 and SW1990 cells (Figure 10E). These results suggested that knockdown of LINC01559 suppressed PC cell proliferation, migration, and invasion *in vitro.*

### Inhibition of LINC01559 Suppressed PC Cell Autophagy and Promotes Apoptosis

**FIGURE 11:**
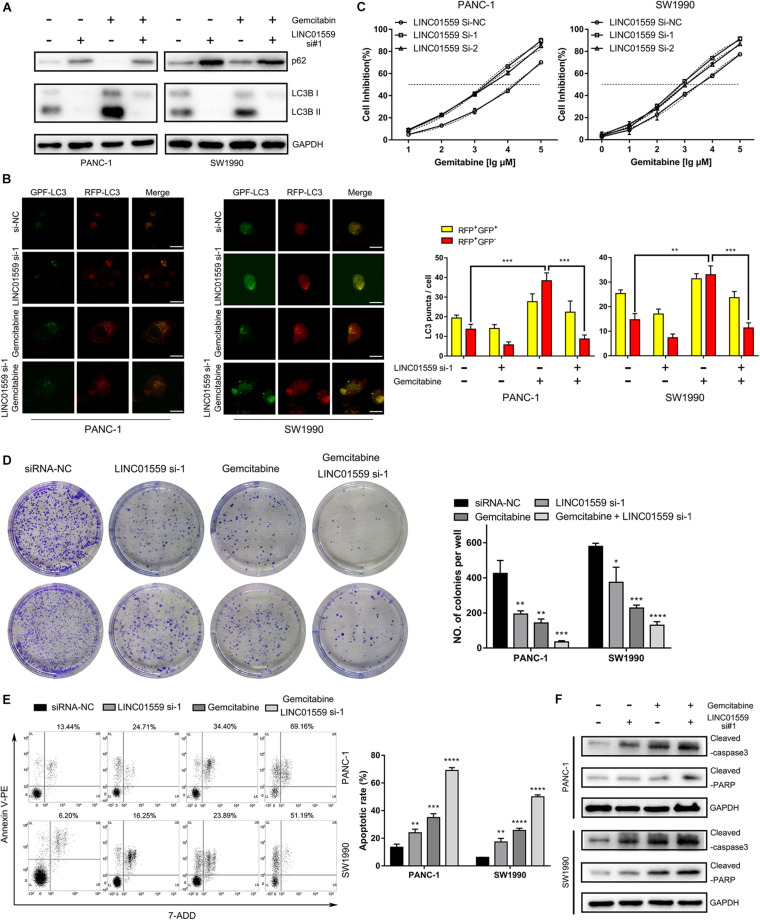
Inhibition of LINC01559 suppressed PC cell autophagy and promoted apoptosis. **(A)** Protein expression levels of p62 and LC3B after various treatments measured by Western blot analysis. **(B)** Representative confocal images of autophagosome (yellow puncta) and autolysosome (red puncta) formation are presented in the left panel. Scale bar: 20μm. The numbers of RFP^+^GFP^+^ LC3 puncta and RFP^+^GFP^–^ LC3 puncta are shown in the right panel. **(C)** The cell inhibition was calculated by the CCK-8 assay in PC cells treated with different concentrations of gemcitabine at 48 h. **(D)** Representative images from the clonogenic assays of PANC-1 and SW1990 cells with or without gemcitabine (10 μM) treatments and cultured for 14 days. **(E)** Apoptosis rate after various treatments was detected by flow cytometry. **(F)** Protein expression levels of cleaved PARP and cleaved caspase-3 after various treatments measured by Western blot analysis (*p < 0.05; **p < 0.01; ***p < 0.001; ****p < 0.0001; ns, not significant).

The role of LINC01559 in autophagy and chemotherapeutic resistance was further explored. WB analysis were employed to show that PC cells transfected with si-LINC01559 exhibited decreased the expression of LC3I/LC3II but increased the p62 expression, indicating that autophagy was inhibited after LINC01559 depletion (Figure 11A). However, gemcitabine (10 μM) treatment induced autophagy in PC cells (Figure 11A). This observation was further confirmed by the tandem LC3B-RFP-GFP fluorescence microscopy assay. As shown in Figure 11B, gemcitabine increased the number of red-only LC3 puncta in PC cells, implying an increase of autophagic flux. Inhibition of LINC01559 reduced the number of red-only LC3 puncta in GFP-mRFP-LC3-transfected PC cells compared with the cells treated with gemcitabine. Besides cell viability assay, colony formation assay, cell apoptosis assay, and WB analysis of apoptotic markers were performed. CCK-8 results showed that the IC50 value for gemcitabine was significantly increased in LINC01559-silenced PC cells (Figure 11C). In contrast, knockdown of LINC01559 significantly induced the colony-forming capacity of PANC-1 and SW1990 cells (Figure 11D) and increased the gemcitabine-induced apoptosis rates (Figure 11E). And the protein level of cleaved caspase3 and PARP were increased in LINC01559-downregulated cells with or without gemcitabine (10 μM) treatment (Figure 11F). These results suggested that the inhibition of LINC01559 could suppress autophagy and stimulate apoptosis, which would ultimately lead to sensitize PC cells to gemcitabine.

## Discussion

PC is a solid tumor with a highly malignant behavior, which has become the fourth largest cancer causing cancer-related death in western countries, with a 5 years survival rate of less than 10% (Siegel et al., 2019). Accumulated evidence showed that autophagy got involved in tumor development and treatment resistance in PC (Piffoux et al., 2020). Thus, it is essential to screen autophagy-related molecular to identify effective prognostic biomarkers for PC. Recent great advances in genomics have provided opportunities for the identification of cancer prognostic biomarkers and potential molecular targets. Here, we were the first to construct a reliable prognostic signature based on autophagy-associated lncRNAs (ARlncRNAs) and confirmed the clinical utility in PC patients. Moreover, we preliminary explored the carcinogenic role of LINC01559 in PC and found that the inhibition of LINC01559 might be a potential therapeutic strategy for improving sensitivity to gemcitabine in PC patients.

Firstly, we employed a Pearson correlation analysis to identify ARlncRNAs, and 826 lncRNAs were obtained. Then, these ARlncRNAs were screened to establish a six-ARlncRNAs signature in training cohort. Next, KM survival analysis and ROC analysis demonstrated the prognostic value of the signature in training cohort. And the similar results were also observed in both validation cohort and entire cohort. Furthermore, the independent prognostic role of the signature was confirmed by the univariate and multivariate cox regression analysis. Moreover, a nomogram indicated a great predictive power of the prognostic signature.

To further explore the clinical utility of the signature, we investigate the association of the signature with clinical features and found that high risk score was positive correlated with tumor grade, AJCC stage, N stage, and T stage. Immune cell infiltration analysis showed the risk score was negatively correlated with plasma cells and CD8 T cells. Moreover, the tumor mutation burden (TMB) of PC patients in the high-risk group was statistically higher than that in the low-risk group, indicating that immunotherapy may be a potentially effective option to those PC patients with high-risk scores. Next, correlation analysis showed that the signature scores were positively correlated with the expression of PD-L1, EGFR, and MET, implying that those PC patients with high-risk scores may be sensitive to these targeted chemotherapy drugs.

To better understand the potential biological mechanism of the involved ARlncRNAs, we constructed the lncRNA-miRNA-mRNA regulatory network. As indicated by KEGG pathway and GO enrichment analysis, the regulatory network may promote therapeutic resistance of PC. And several pathways, such as Toll-like receptor signaling pathway, ErbB signaling pathway, and hedgehog signaling pathway, may be involved in the process.

Most importantly, we identified six potential small molecule drugs from the network, including vorinostat, trichostatin-A, sirolimus, phthalylsulfathiazole, GW-8510, and daunorubicin. Vorinostat is a histone deacetylase (HDAC) inhibitor approved by FDA for the treatment of patients with refractory or relapsed cutaneous T cell lymphoma. Pre-clinical studies have demonstrated that vorinostat could induce apoptosis and inhibit tumor growth in human PC cell lines. And the combination of vorinostat and capecitabine with radiation in PC patients were well tolerated with antitumor activity in a phase I clinical trial (NCT00983268) (Chan et al., 2016). Trichostatin A (TSA), a natural derivative of dienohydroxamic acid, is a potent inducer of tumor cell growth arrest, differentiation, and apoptosis. Donadelli et al. (2003) demonstrated the antitumor value of TSA in human PC cell lines. And, combined therapy of gemcitabine and TSA exerted synergistic inhibition of PC cell growth (Donadelli et al., 2007). Sirolimus, also called rapamycin, is an immunosuppressive agent proved by FDA mainly for the prophylaxis of organ rejection in patients receiving renal transplants. However, as a derivative of sirolimus, everolimus exerts anti-angiogenic properties and is indicated as the first line therapy for pancreatic neuroendocrine tumor. And, clinical trials of everolimus in combination with other anticancer agents in PC patients is going on. Phthalylsulfathiazole is a broad-spectrum antimicrobial agent which is used in the treatment of dysentery, colitis, and gastroenteritis. It has not been reported that the agent shows anti-tumor effects. GW8510 is a synthetic cyclin-dependent kinase (CDK) inhibitor that could reverse tamoxifen resistance in breast cancer and gemcitabine resistance in lung squamous cell carcinoma through autophagy induction (Chen P. et al., 2018; Li et al., 2020). Daunorubicin is the first generation of anthracyclines with antineoplastic activity and is indicated exclusively for the treatment of acute leukemia. Anthracycline drugs, including daunorubicin, doxorubicin, epirubicin, therarubicin, and aclacinomycin, are widely used in the treatment of hematologic malignancies and solid tumors. Taken together, these findings provide potential therapeutic options for patients with PC.

Among the six selected ARlncRNAs, LINC01559 was reported to be upregulated in renal cell carcinoma, gastric cancer, PC, and hepatocellular carcinoma (Chen B. et al., 2018; Chen et al., 2020; Dong et al., 2020; Lou et al., 2020; Wang et al., 2020). Moreover, we found that the expression of LINC01559 was significantly increased in both serum and tumor tissues of PC patients, indicating that LINC01559 could serve as a diagnostic biomarker. Thus, we chose LINC01559 for further analysis. Functional analysis showed inhibition of LINC01559 suppressed proliferation, migration, and invasion in PANC-1 and SW1990 cells. The results were similar to Lou et al. (2020) and Chen et al. (2020) in AsPC-1, BXPC-3, MIA PaCa-2 cells. Interestingly, Dong et al. (2020) reported that LINC01559 may be involved in regulating the resistance and response to oxaliplatin in hepatocellular carcinoma. Then, we investigated the relationship of LINC01559 expression and chemoresistance. *In vitro* analysis showed that silencing LINC01559 suppressed the gemcitabine-induced autophagy and promoted gemcitabine-induced apoptosis, implying that inhibition of LINC01559 could be a potential therapeutic treatment for improving sensitivity to gemcitabine in PC patients.

Although there have been many reports of bioinformatic analysis of PC (Wei et al., 2019), we focused on the essential role of autophagy-related lncRNAs (ARlncRNAs) in biological characteristics of tumor malignancy and first proposed a six-ARlncRNAs signature for PC cohort. Moreover, we validated the independent prognostic value of the signature and explored in depth the clinical utility for predicting efficacy of immunotherapy and targeted therapy in PC patients. More importantly, we constructed an lncRNA-miRNA-mRNA regulatory network to better understand the potential biological mechanism. And, cMAP analysis was performed to screen potential small molecule drugs for patients with PC, which may provide clinical benefits. However, there are inevitably several limitations in our paper. First, due to the lack of valid data, our prognostic model and nomogram cannot be verified by external data. Second, the universality of the conclusion may be limited by the influences of race/ethnicity in PC patient TCGA cohorts. Moreover, despite the reports that LINC01559 regulate proliferation and migration by acting as a competing endogenous RNA of miR-1343-3p and miR-607 (Chen et al., 2020; Lou et al., 2020), the biological role of LINC01559 in regulating autophagy is obscure. Thus, we should combine multicenter clinical trials and prospective study to further prove the clinical value of the model in PC and it’s essential to further elucidate the molecular mechanisms that link LINC01559 with autophagy.

In summary, our study provided a deeper understanding of the role of autophagy in biological characteristics of tumor malignancy and firstly proposed a six-ARlncRNAs signature that could provide valuable clinical utility for reliable prognostic prediction and personalized treatment of PC patients. Moreover, we identified the prognostic role of LINC01559 in PC, and targeting LINC01559 may be a potential therapeutic option for overcoming the resistance to gemcitabine in PC patients.

## Data Availability Statement

The raw data supporting the conclusions of this article will be made available by the authors, without undue reservation.

## Ethics Statement

The studies involving human participants were reviewed and approved by the Ethics Committee of the Tongji Hospital, Tongji Medical College, Huazhong University of Science and Technology. The patients/participants provided their written informed consent to participate in this study.

## Author Contributions

ZD and XL designed the study. XL, ZD, and YS completed the experiments and analyzed the data. XL, ZD, and YL wrote the manuscript and were responsible for language revisions. WY and JW supervised the project. All authors reviewed the manuscript.

## Conflict of Interest

The authors declare that the research was conducted in the absence of any commercial or financial relationships that could be construed as a potential conflict of interest.

## Footnotes

1 https://portal.gdc.cancer.gov

2 http://asia.ensembl.org/index.html

3 http://autophagy.lu/clustering/index.html

4 http://www.gencodegenes.org

5 http://caret.r-forge.r-project.org/

6 http://carolina.imis.athena-innovation.gr/diana_tools/web/index.php?r=lncbasev2%2Findex

7 http://mirdb.org/miRDB/

8 http://mirtarbase.mbc.nctu.edu.tw/

9 http://targetscan.org/

10 https://cytoscape.org/

11 https://portals.broadinstitute.org/cmap/

12 https://pubchem.ncbi.nlm.nih.gov/

13 http://gepia2.cancer-pku.cn/

## Abbreviations

PC: pancreatic carcinoma
lncRNA: long non-coding RNA
ARlncRNA: autophagy related lncRNA
TCGA: The Cancer Genome Atlas
DEGs: differentially expressed genes
GO: Gene Ontology
KEGG: Kyoto Encyclopedia of Genes and Genomes
ROC: receiver operating characteristic.
